# A Distributed VMD-BiLSTM Model for Taxi Demand Forecasting with GPS Sensor Data

**DOI:** 10.3390/s24206683

**Published:** 2024-10-17

**Authors:** Hasan A. H. Naji, Qingji Xue, Tianfeng Li

**Affiliations:** School of Digital Media, Nanyang Institute of Technology, Chang Jiang Road No. 80, Nanyang 473004, China; xue_qj@sina.com (Q.X.); ltf@nyist.edu.cn (T.L.)

**Keywords:** taxi demand forecasting, GPS sensor data, variational mode decomposition, spark platform, bidirectional long short-term memory

## Abstract

With the ubiquitous deployment of mobile and sensor technologies in modes of transportation, taxis have become a significant component of public transportation. However, vacant taxis represent an important waste of transportation resources. Forecasting taxi demand within a short time achieves a supply–demand balance and reduces oil emissions. Although earlier studies have forwarded highly developed machine learning- and deep learning-based models to forecast taxicab demands, these models often face significant computational expenses and cannot effectively utilize large-scale trajectory sensor data. To address these challenges, in this paper, we propose a hybrid deep learning-based model for taxi demand prediction. In particular, the Variational Mode Decomposition (VMD) algorithm is integrated along with a Bidirectional Long Short-Term Memory (BiLSTM) model to perform the prediction process. The VMD algorithm is applied to decompose time series-aware traffic features into multiple sub-modes of different frequencies. After that, the BiLSTM method is utilized to predict time series data fed with the relevant demand features. To overcome the limitation of high computational expenses, the designed model is performed on the Spark distributed platform. The performance of the proposed model is tested using a real-world dataset, and it surpasses existing state-of-the-art predictive models in terms of accuracy, efficiency, and distributed performance. These findings provide insights for enhancing the efficiency of passenger search and increasing the profit of taxicabs.

## 1. Introduction

In modern urban cities, taxicabs and transportation network companies provide reliable and convenient services to a huge number of passengers. The availability of reliable GPS sensor data plays a vital role in understanding taxi operations, facilitating the development of advanced models and algorithms, and supporting data-driven decision-making processes in the transportation industry. The ridership of transportation network companies has been rapidly increasing. For instance, from 2017 to 2021, Uber’s trips grew from 3.79 billion to 6.3 billion [1]. Hence, there is a pressing need to develop robust models that effectively utilize taxi trajectory data to enhance prompt passenger retrieval by taxis. Taxi demand forecasting plays an essential role in taxi services pre-allocation. Enhancing taxi demand forecasting improves the efficient use of urban transportation systems by keeping a balance between taxicab resources and the demand for pre-allocated resources. Moreover, accurate prediction provides high-efficiency management for urban traffic and urban transportation systems [2]. In the field of self-driving, ride-sharing services can benefit from taxi demand prediction to enlarge the transportation capacities of cities and have a significant impact on human travel rates and transport modes [3].

Two challenges are encountered in taxi services: (1) the number of vacant taxis may decrease taxi utilization, and (2) the satisfaction of waiting passengers may decrease. These issues can influence taxi operators and ride services. Therefore, improving short-term taxi demand prediction becomes a challenging and essential issue. Short-term taxi demand prediction can provide insights into the understanding of taxi demand distribution in areas of urban cities, and then more accurate taxi demand forecasting can decrease the waiting time of passengers. As a result, we can improve the taxi utilization rate and raise taxi services’ profitability and transportation efficiency.

Considerable research efforts have been dedicated to the field of taxi demand prediction, resulting in the proposal of numerous forecasting methods. Traditional investigations predominantly employ empirical statistics and machine learning techniques to tackle this challenge [4,5]. These approaches have shown substantial improvements in prediction accuracy and have garnered increasing attention from researchers [6]. Faghih et al. [7] presented an innovative approach for forecasting short-term Uber service demands in New York City using a spatial–temporal autoregressive model. Spatial and temporal patterns are considered for forecasting taxi requests. In [8], GPS streaming data are utilized to predict 30 min taxi demands using Poisson and ARIMA methods. An approach introduced by [9] incorporates a clustering method and ordinal regression models for conducting taxi demand and trip price prediction.

Various machine learning-based methods have been applied to taxi demand forecasting. For instance, Guo et al. [10] integrated a backpropagation network and an XGBoost to predict online demand for taxis. In [11], a machine learning-based method is proposed for predicting ride-hailing taxi demand. In [12], a multiple tasks learning-based model combined with three LSTM models is proposed to forecast pickup taxi demand. In [13], the authors present a hyped method for taxi passenger prediction using an integration of a Convolutional Neural Network (CNN) and a Long Short-Term Memory network (LSTM). In the model, the CNN is applied to capture the spatial features, and the LSTM network is utilized to capture the temporal features. Xia et al. [14] introduced the WNDLSTM model as a parallel long short-term memory weighted model integrated and applied in the MapReduce framework. In [15], a traffic demand prediction model is proposed by integrating a Convolutional Neural Network (CNN) and Long Short-Term Memory (LSTM) to extract spatiotemporal features and capture and examine their impact on ride-hailing demands. A taxi demand prediction method named Multi-Level Recurrent Neural Networks (MLRNNs) [16] is put forward to explore the main features shared among taxi zones and extract the unique features of taxi zones. In [17], a distributed Grid-search-aware Support Vector Machine is introduced to address the issue of passenger hotspot prediction.

Although the taxi demand prediction problem has been addressed in the aforementioned studies, performing accurate and efficient taxi-demand prediction is still tricky, and three significant issues can be found: (1) The non-linear and non-stationary features of taxi data. The generated GPS data are different and complicated characteristics in various traffic conditions in other geographic areas and periods. Therefore, some approaches apply EMD-based algorithms [18,19], and some other design interpretability index and perform partial dependence analysis [20]. However, there is still a challenge in looking for models that can efficiently deal with non-linear and non-stationary features and provide accurate predictions for taxi demands and traffic flow. In this study, Variational Mode Decomposition (VMD) is applied to decompose time series datasets into sub-series to reduce the influence of non-linear and non-stationary features of taxi data. (2) The existence of gradient explosions, the issue of gradients growing bigger and bigger, gradient disappearance, the issue of gradients becoming smaller and smaller and approaching zero in forecasting methods, and the inability to extract time series features for specific short periods present challenges. Previous studies utilized machine learning models such as RNNs, LSTM, and GRU [14,18]. Although these models are well suited for modeling sequential dependencies, which are vital in traffic prediction, the BiLSTM mode is capable of utilizing information from both sides that remarkably increases the accuracy and solves the gradient disappearance issue. Therefore, BiLSTM is used in this study. (3) The traffic data are constantly increasing, and with the exponential growth of these data, existing algorithms suffer from certain limitations on the centralized mining platform. Many issues still emerge when dealing with large-scale datasets, and training predictive models is time-consuming and requires massive computational resources, leading to low prediction performance. Although some researchers [14,21] adopted Hadoop’s MapReduce platform for high-performance computation, the computation load was still relatively huge and requires enhancement. Our study adopted a Spark parallel framework, which can be 100 times faster than MapReduce, by applying Resilient Distributed Dataset (RDD) operators, which eliminate the need to store intermediate files, and by this method, the accuracy, efficiency, and robustness of the traffic data are highly improved.

To address the above challenges, we propose a novel distributed model for predicting taxi demands named VMD-BiLSTM. It integrates the Variational Mode Decomposition (VMD) method and the Bidirectional LSTM (BiLSTM) model to perform the feature extraction and the prediction of taxi demand, respectively. Taxi demand data are decomposed using the VMD method to generate intrinsic modal functions (IMFs), thereby reducing the non-stationarity of the taxi demand data. Bidirectional LSTM (BiLSTM) is utilized to perform the forecasting process as its ability to capture both preceding and following features result in a remarkable increase in the overall model accuracy. The proposed model is deployed on Apache Spark to conduct the time series prediction of industry big data utilizing parallelization technology. Using the VMD-BiLSTM model, taxi demand can be forecasted in a way that significantly reduces computation time while maintaining high accuracy in a distributed manner. To sum up, this study makes several important contributions:

(1) This study proposes a hybrid prediction model (VMD-BiLSTM) for taxi demand prediction by utilizing the VMD algorithm for dealing with the non-linearity and the degradation of prediction accuracy in time series data, along with a BiLSTM model for capturing both forward and backward information to improve the forecasting accuracy.
(2) The model conducts taxi demand prediction on the Spark distributed platform to benefit from the advantages of parallel processing to address time computation costs and achieve high scalability.
(3) The proposed model is tested using a real-world GPS dataset obtained from taxicabs in Wuhan city, and the performance of VMD-BiLSTM is compared with four cutting-edge algorithms. The findings show that the model can outperform similar prediction models in terms of accuracy, scalability, and efficiency.

The rest of this article is structured as follows. Section 2 introduces the problem of demand prediction. The proposed methodology, including the data preprocessing and the distributed VMD-BiLSTM model, is presented in detail in Section 3. In Section 4, we described the experimental settings, and compared models and the evaluation metrics. Section 5 presents the obtained results and a detailed analysis of the implications, contributions, and limitations of this study in the dissection part in Section 6. Finally, Section 7 summarizes the conclusions of this study and highlights directions for future research.

## 2. Problem Statement

Forecasting taxi demand is commonly considered a problem for time series prediction. Specifically, suppose historical data of taxi demand are represented as D_t−s_,D_t−s+1_, …, D_t−1_. The objective is to predict the next taxi demand, for the *t*th interval, expressed as follows:

D_pred_ = *F*(D_t−s_, D_t−s+1_, …, D_t−1_) (1)

where *s* represents the total steps of historical times, whereas *F* is the forecasting function. The forecasting function *F* is fed by the historical data as input and then generates the predicted value as the function’s output. The initial objective of this research is to produce a forecasting function that can generate predictions with a relatively high level of accuracy while maintaining acceptable time consumption and stability. This aim is pursued by utilizing a substantial volume of taxi GPS trajectories as the input data for the function. By leveraging the rich information contained in these trajectories, this study aims to capture the intricate patterns and trends related to taxi demand.

Accurate prediction plays a crucial role in various processes in the taxi industry, such as resource allocation, route planning, and service optimization. Therefore, it is essential to develop a function that can effectively analyze the complex dynamics of taxi demand and provide reliable predictions. Achieving both accuracy and efficiency is particularly important in real-world applications where timely responses are required.

Moreover, the function’s stability is a critical factor to ensure consistent and dependable predictions over time. By considering a massive number of taxi GPS trajectories, this study aims to enhance the robustness and stability of the forecasting function to address various scenarios and adapt to potential changes in taxi demand patterns.

Overall, this study aims to contribute to the field of taxi demand prediction by developing a forecasting method that strikes a balance between accuracy, time consumption, and stability. The findings of this research can have practical implications for improving operational efficiency and decision-making processes in the taxi industry.

## 3. Distributed VMD-BiLSTM Forecasting Model

**Figure 1:**
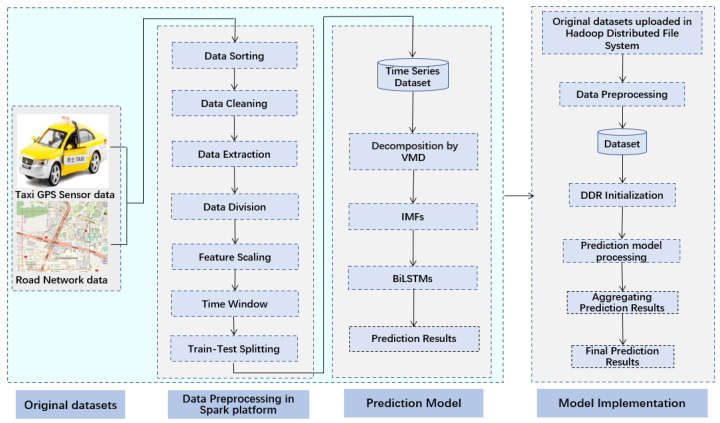
The structure of the distributed VMD-BiLSTM prediction model.

Figure 1 provides an overview of the VMD-BiLSTM model employed in this study, consisting of three distinct modules, namely, original datasets, data preprocessing, and the prediction model. These modules operate synergistically to accomplish the objective of precise and efficient prediction of taxi demand.

### 3.1. Original Datasets

In this study, two types of datasets are operated, namely the Taxi GPS sensor dataset and the Road network dataset.

#### 3.1.1. Taxi GPS Dataset

The taxi GPS sensing dataset, as collected by traffic sensor centers and utilized for this study, was obtained from 9124 GPS-enabled taxis operating in the city of Wuhan, China, over 122 days, approximately four months. On average, each taxicab generated more than 3500 GPS sensor records/day, and all the vehicles used in this study resulted in around 320 million records. Each GPS record contains the following attributes: Taxi Identifier, Timestamp, Location Coordinates, Vehicle Speed, Taxi Occupy Status, and Vehicle Direction.

#### 3.1.2. Road Network Dataset

**Figure 2:**
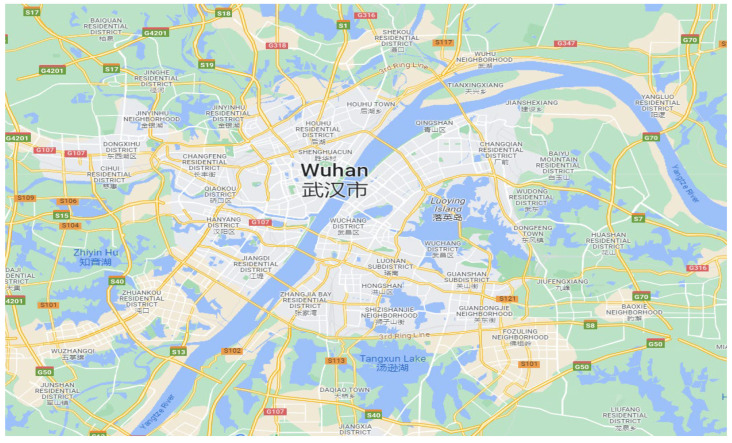
Road map network of Wuhan City.

The road dataset utilized in this study was obtained via the OpenStreetMap. A simplified map of the road network of Wuhan City, China, is presented in Figure 2. The map gives a visual representation of the interconnected road segments and intersections in the city.

The road network dataset provides a comprehensive collection of information regarding the road network. It encompasses a vast amount of data, including 95,781 road segments and 94,214 intersections. Additionally, the dataset includes the precise latitude and longitude coordinates associated with each intersection and road segment. These coordinates offer valuable insights for various applications and analyses related to the road network.

### 3.2. Data Preprocessing

The original raw datasets contain errors due to various factors such as GPS equipment failures, errors in taxi driver operations, and signal delays. These errors can result in issues such as inaccurate or inconsistent data. Additionally, to ensure the reliability and accuracy of the proposed model used for taxi demand prediction, preprocessing the raw data is necessary to address the incorrect data and the invalid data, extract the main features, and reform the data in a way that makes them applicable to the prediction model. All the steps involved in preprocessing are shown in Figure 1.

It is important to mention that the steps of data preprocessing were distributedly performed on the Spark platform. The target raw dataset was uploaded to HDFS (Hadoop Distributed File System), which provides distributed storage for big datasets whereas the Spark platform (using the pyspark package in Python) provides a Resilient Distributed Dataset (RDD) file splitting technique. An RDD uses the map transformation method to apply a function to each element in an RDD to divide the rows of an RDD file by a delimiter, and then a fast processing technique is conducted to increase data preprocessing speed and to ensure the scalability and accuracy of the prediction model.

#### 3.2.1. Data Sorting

The original GPS trajectory data were collected by periodically sending signals that described taxi information at a 30 s interval to produce a massive dataset. The massive dataset was firstly stored on the Hadoop Distributed File system platform (HDFS), and a sorting step for the dataset records was required. A partition-based sorting operation was then utilized for its ability to sort records without shuffling data across the Hadoop cluster. The *sortWithinPartitions* method in Spark was applied on the timestamp column; in general, this may not result in a whole dataset featuring globally sorted column. Each partition was locally sorted as listed in Table 1a. After that, the *sortWithinPartitions* method was applied to the TaxiID column, and then the sorted dataset could provide a more comprehensive analysis of the trajectories for individual taxis. The sorted dataset is depicted in Table 1b, visually demonstrating the organized and grouped trajectories for each taxicab.

#### 3.2.2. Data Cleaning

The geographical coordinates of Wuhan city were considered, and all taxi GPS records located outside these coordinates were removed. Additionally, certain criteria were applied to filter out specific GPS data points. Specifically, data points with a taxi status of 0 or 2, where 0 represents an invalid device and 2 indicates a temporarily stopped taxi, were excluded from the sensing dataset. The remaining records were stored in a filtered RDD and duplicate records and records with null values were further removed from the dataset.

Technically, the content of the dataset was loaded into a big-size dataframe which consisted of a huge number of rows and columns. After loading the dataset, a basic exploration process was performed to understand its structure, schema, and contents using the dataframe, which consisted of many NULL or None values at some rows or columns. Therefore, rows with missing values were dropped via the *dropna*() function, and duplicate rows were removed using *dropDuplicates*(). Moreover, outliers were identified and removed based on statistical measures such as z-score, standard deviation, or percentiles. For example, by applying the *Filter*() function, rows were filtered based on a condition.

#### 3.2.3. Data Extraction

**Figure 3:**
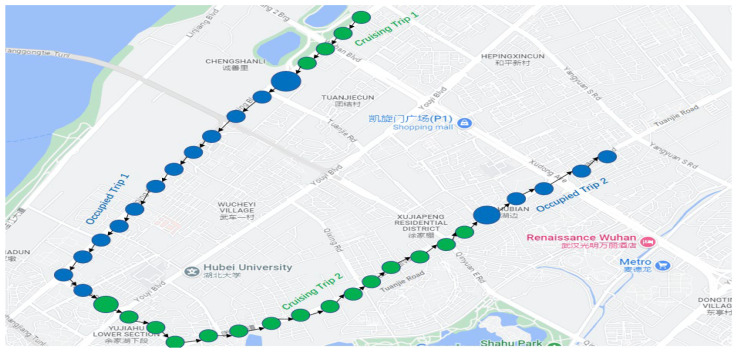
Typical trajectory of taxi trips.

After completing the data cleaning process, the next step was to extract information about taxi trips, i.e., when taxicabs were cruising the city roads with passengers. This information could be obtained by analyzing the taxi status recorded in the GPS sensor dataset. Specifically, if the taxi status of a taxicab is 1, it indicates that no passenger is in the taxi at that moment. On the other hand, once the taxi status is 3, it signifies that the taxi is occupied with passengers, as depicted in Figure 3.

The visualization in Figure 3 represents taxi trips using small green circles to indicate cruising trips and small blue circles to indicate occupied trips, whereas a large circle represents the event when a passenger is picked up by a taxi, resulting in the taxi’s occupied status, or a passenger drop-off from a taxi, wherefore a cruising trip begins. Table 2 describes the attributes of an occupied trip.

By isolating occupied trips, the subsequent analysis and prediction models can accurately capture the patterns and trends in the taxi demand of passengers [22].

For the sake of enhancing the accuracy and reliability of the dataset, additional refinement was performed. Given the scale of the dataset, a specific large study area within the city of Wuhan, known as the Wuchang district, was selected for focused analysis. Wuchang district was chosen because of its relevance and significance to this study.

**Figure 4:**
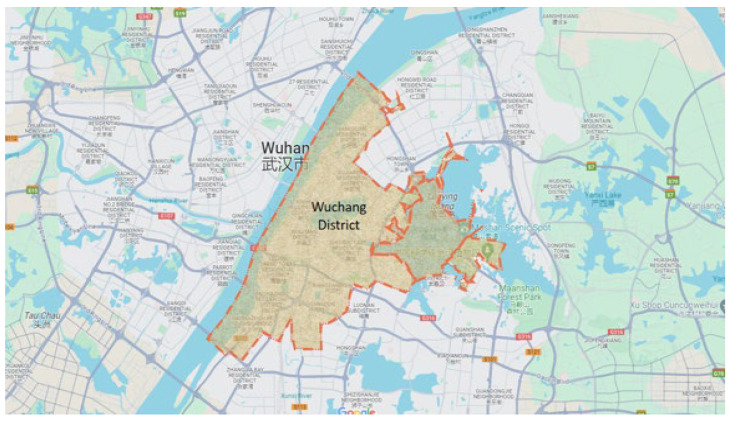
Study area of Wuchang district.

Figure 4 displays the road network map of the Wuchang district, providing a visual representation of the road infrastructure within the selected study area.

The selection of the Wuchang district as the focus of analysis was driven by two factors. Firstly, this area exhibits a significant volume and distribution of taxi trips, making it a representative sample for studying taxi demand. Secondly, a comprehensive analysis of the Points of Interest (POI) dataset revealed that the area is identified by various establishments, including universities, shopping malls, hospitals, business companies, and more. The extracted trip dataset specifically for the Wuchang district comprised approximately 1,421,935 records.

Technically, the required rows were extracted and processed using the filter function by adding the related conditions. For instance, once trip information was required, rows were filtered by obtaining the rows within the taxi status between 3 (pickup event) and 1 (drop-off event); each group of such records was represented as an occupied trip.

#### 3.2.4. Data Division

**Figure 5:**
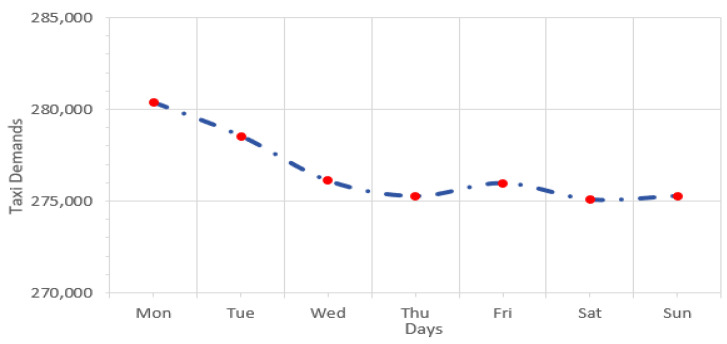
The distribution of taxi demands on the weekdays and weekends.

To focus on the targeted study area, the taxi trips within the Wuchang district area were processed to retain only the temporal features. Then, the distribution and the temporal features of taxi demands were extracted. Figure 5 illustrates the distribution of taxi demands on weekdays and weekends in the study area.

**Figure 6:**
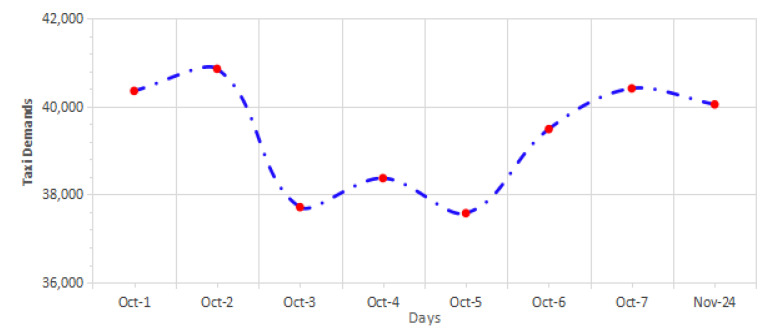
Taxi demand distribution in the target area during holidays.

Regarding the distribution of taxi demand on holidays, the gathered dataset included eight days (Chinese National day holiday on 1–7 October and 24 November for Christmas day). The distribution of taxi demand on holidays for the target area is illustrated visually in Figure 6.

A remarkable pattern of the demand trends during holidays is presented in Figure 6. The days of the 1st and 2nd of October present a high rise in taxi demands which is followed by a steady decrease. A slight fluctuation is found in the subsequent days, and eventually taxi demands had another high value on the 7th of October. This indicates that there was a slight increase in taxi demand at the end of the holiday period. At the beginning of the October holiday period, and on 24 November, people take taxis to travel. Additionally, taxi demands dramatically rise on 7 October as people come back after a holiday.

**Figure 7:**
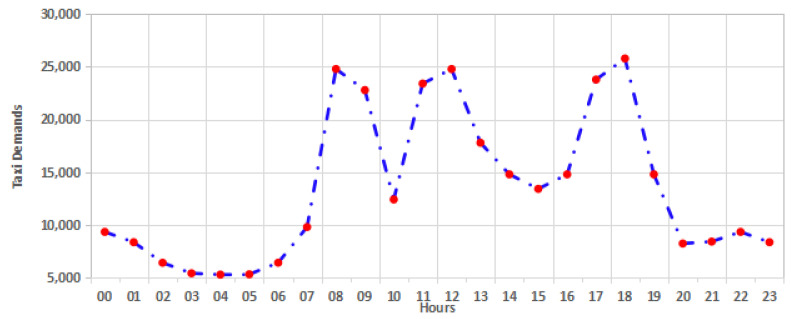
The distribution of taxi demands in the target area over 24 h.

Figure 7 shows the average distribution the taxi demands over 24 h in the target area in Wuhan City, China.

There are some obvious trends in the study area. For instance, the minimum rates are at 3:00, 4:00, and 5:00, whereas the peak rush is at 8:00, 9:00, 11:00, 12:00, 17:00, and 18:00. The values of the demands contain high periodicity over the long term, and the demand numbers on Saturdays and Sundays are quite similar. The data division process was applied on the Spark platform through a *filter*() function by limiting timestamps with required dates and hours.

#### 3.2.5. Feature Scaling

Feature scaling is an essential step in data preprocessing to ensure that the independent variables or features of the data are normalized and comparable [23]. There are several main scaling methods such as the Clipping method, Standard Deviation method, Min/Max, and Z-score. The latter two methods are popular in use. The Min/max method transforms the minimum value of a feature into 0 and the maximum value transforms it into 1. Z-score is another scaling method, which is better used in numerical values in multiple dimensions with significant differences in size; Z-score was suitable for our taxi prediction as the time windows of the dataset could sometimes have extremely high demands and sometimes have very low demands. Therefore, in this study, the feature scaling process was performed using the Z-score method, which is computed via Formula (2):

Z = (*x* − *μ*)/*σ* (2)

where *x* represents the original value, and the mean and the standard deviation of the dataset are denoted by *μ* and *σ*, respectively.

The result of applying the Z-score method on the target dataset is that each feature value is rescaled with the distribution value between 0 and 1 is useful for the prediction models feeding with weight inputs to assist in enhancing the prediction findings.

#### 3.2.6. Time Window

In this study, the aim was to utilize the spatial information of the target region in conjunction with the corresponding taxi demands to generate predictions for a specified time window. Through an analysis of the distribution of taxi demands, it was observed that certain hours exhibit significantly higher taxi demand levels.

Following the time window designing method in [24], a time window set can be generated from the dataset by periods as follows: *t*_−7_, *t*_−6_, *t*_−5_, *t*_−4_, *t*_−3_, *t*_−2_, *t*_−1_, and *t*, where each time interval period can be defined by exploring the optimal prediction horizon (can be 5, 10, 15, or 20 min). The time window step resulted in taxi demands for the time intervals which later would be the inputs fed into the proposed model to attain the prediction results for the next period *t*_+1_.

Spark performs the time window splitting *window*() function by determining the start and end time for each time window, by default using the nested columns representing the “start” and “end” attributes. The *RangeBetween*() function is another function for obtaining more accurate results of time window ranges.

#### 3.2.7. Train Test Data Splitting

For the aim of simplifying the training and the evaluation of the applied models, the dataset was divided into training and testing datasets. This division was carried out using a ratio of 30/70, where 70% of the data were allocated for the training step, and the remaining 30% were reserved for the testing and evaluation steps.

Technically, for the aim of splitting the dataset into a training and test set in PySpark, the *randomSplit*() function was applied by setting the weights argument, which specifies the percentage of samples from the dataset that must be placed in the training set (70%) and test set (30%), respectively.

### 3.3. VMD-BiLSTM Model

Traffic data are subject to the influence of spatial and temporal factors characterized by essential non-linearity, uncertainty, and temporal instability, challenging the accuracy of traditional forecasting models. Therefore, this study designed a novel hybrid model integrating the VMD algorithm along with the BiLSTM model to enhance traffic prediction accuracy. Initially, the Variational Mode Decomposition (VMD) algorithm was applied to extract the intrinsic mode function of the time series data, enabling a more accurate exploration of the signal’s dynamic features.

**Figure 8:**
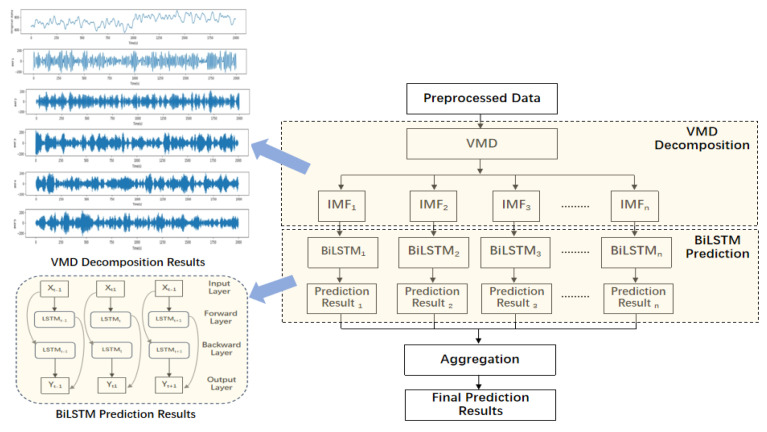
Schematic diagram of the VMD-BiLSTM model.

VMD technology adeptly deals with non-linear and non-stationary signals, circumventing the issues of spurious components and edge effects, showing exceptional signal processing abilities. BiLSTM explores information from both the past and future at specific time intervals. The bidirectional approach allows the network to capture features more fully and improve its predictive performance. By merging the advantages of the data decomposition method and a deep learning model, the hybrid model presented in this study can significantly enhance the accuracy and reliability of taxi demand prediction. The overall diagram of the proposed model is shown in Figure 8.

Before delving into the details of the proposed VMD-BiLSTM model, it is important to provide a concise overview of the VMD (Variational Mode Decomposition) and Bidirectional LSTM (Long Short-Term Memory) methods.

#### 3.3.1. Variational Mode Decomposition

A Variational Mode Decomposition method is an adaptive signal decomposition method proposed by Dragomiretskiy and Zosso [24]. VMD utilizes a non-recursive base signal decomposition method. The VMD algorithm decomposes the target signal into K Intrinsic Mode Functions and then obtains a mean frequency of each IMF. Consequently, a mode u_k_ fluctuates around its respective mean frequency ω_k_.

**Figure 9:**
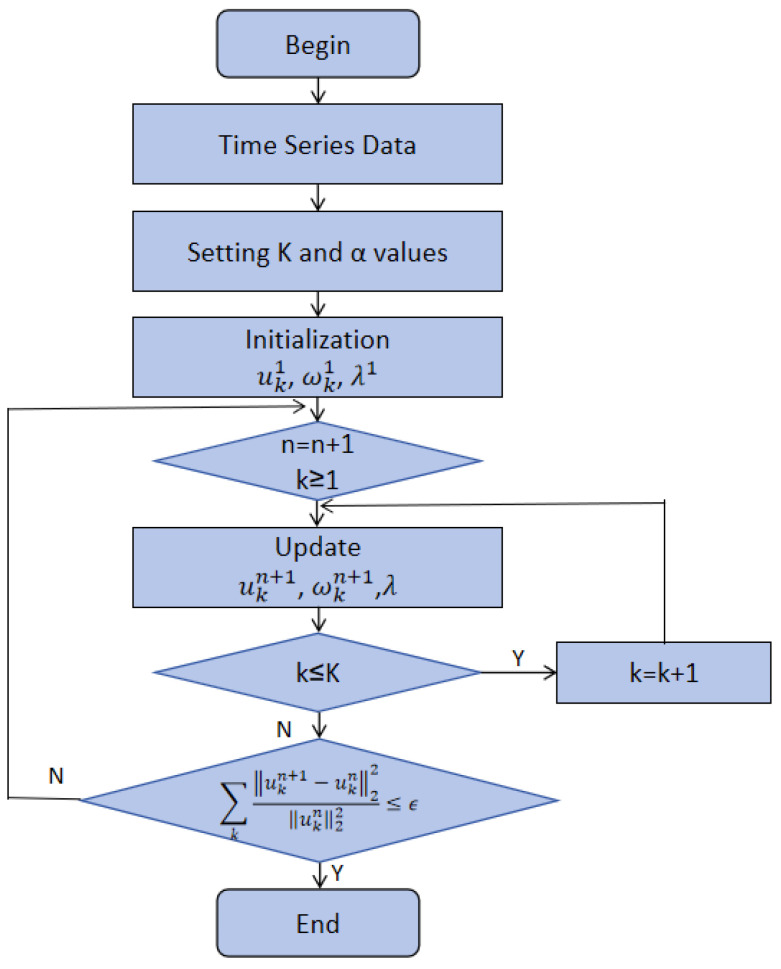
Flowchart of VMD algorithm.

The VMD algorithm utilizes a frequency division process that considers the characteristics of the given signals and continuously updates each intrinsic mode function (IMF) and their central frequency. A flowchart of the VMD algorithm is shown in Figure 9.

By considering a factor α along with a Lagrangian multiplier λ(t), we can transform the complex constrained variational problem into a simpler unconstrained variational problem. The transformation process *L* considers $\left\{u_{k}\right\},\;\;\left\{\omega_{k}\right\},\;\;and\;\lambda$. Then, the values of $\left\{u_{k}\right\},\;\;\left\{\omega_{k}\right\},\;and\;\lambda$ are required to be updated. The update process follows the equations provided below [24]:

(3) $$\omega_{k}^{n+1}\leftarrow \arg_{u_{k}}minL(\left\{u_{i<k}^{n+1}\right\},\left\{u_{i\geq k}^{n}\right\},\left\{\omega_{i}^{n}\right\},\lambda^{n})$$

(4) $$\omega_{k}^{n+1}\leftarrow \arg_{\omega_{k}}minL(\left\{u_{i}^{n+1}\right\},\left\{u_{i<k}^{n+1}\right\},\left\{\omega_{i\geq k}^{n}\right\},\lambda^{n})$$

(5) $$\lambda^{n+1}\left(\omega \right)=\lambda^{n}+\tau \left(f-\sum_{k}u_{k}^{n+1}\right)$$

where the *k* value is in the range [1, K]. In this study, the decomposition process of taxi demand data was conducted using the VMD algorithm.

There are three main parameters considered in VMD and their values play a significant role in the results of the outputs of VMD, namely the Number of Modes K, Penalty Parameter α, and Convergence Tolerance τ. The main challenge in VMD parameter optimization is the trade-off between over-fitting (too many modes or loose regularization) and under-fitting (too few or too tight regularization).

The grid search method was applied to optimize the parameters K, α, and τ of VMD by systematically exploring different combinations of these parameters within specified ranges, selecting the best performance according to a chosen evaluation metric, such as the MSE, and exploring the distribution of the modes resulting from VMD. To be specific, firstly, we defined a range for the key parameters. K ranged from 2 to 7, α ranged from 100~3000, and τ had the range 10^−6^~10^−3^. Then, we analyzed the MSE results to select the optimal combination of the three parameters where the lower the value of the MSE, the better the selection.

Moreover, taxi demand data usually have both temporal and spatial correlations. The temporal correlation of taxi demands implies that a given area is correlated to the temporal variation.

For capturing the spatiotemporal correlation of taxi demand within the increasing amount of data, a two-dimensional (2D) matrix was utilized to place the spatiotemporal features of taxi demands. Suppose there is an array of N columns and M rows on a spatial area that appears as an M × N grid. Using the array, we can index each position by a 2D coordinate (i,j) (1≤i≤M and 1≤j≤N), and each position can be represented by x(i,j)_t_, where M and N donate the indexes of rows and columns, respectively.

Therefore, A one-dimension time series of taxi demands can be represented by a two-dimensional (2D) matrix as follows:

(6) $$x_{t}=\left[\begin{array}{cccc} x(1,1{)}_{t} & x(1,2{)}_{t} & \cdots & x(1,N{)}_{t}\\ x(2,1{)}_{t} & x(2,2{)}_{t} & \cdots & x(2,N{)}_{t}\\ \vdots & \vdots & \vdots & \vdots \\ x(M,1{)}_{t} & x(M,2{)}_{t} & \cdots & x(M,N{)}_{t} \end{array}\right]$$

**Figure 10:**
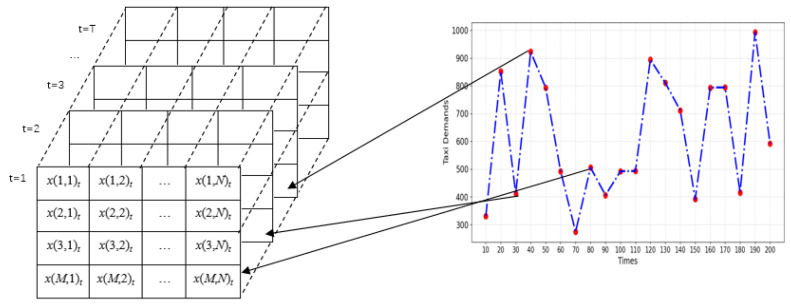
The transformation of the taxi demands time series into a two-dimensional array.

The transformation of the taxi demands time series into a two-dimensional (2D) array is depicted in Figure 10.

The original time series of taxi demands of a spatial site (M, N), which is illustrated by the right graph, can be transformed and presented in the corresponding cell in the matrix on the left graph. The matrix is then decomposed by VMD and prepared for taxi demand prediction. Suppose T represents the time window length and K is the number of IMF components. At a time t, we can represent the value of the site and the values in the matrix after VMD as follows:

(7) $$x_{t}^{k}=\left[\begin{array}{cccc} x(1,1{)}_{t}^{k} & x(1,2{)}_{t}^{k} & \cdots & x(1,N{)}_{t}^{k}\\ x(2,1{)}_{t}^{k} & x(2,2{)}_{t}^{k} & \cdots & x(2,N{)}_{t}^{k}\\ \vdots & \vdots & \vdots & \vdots \\ x(M,1{)}_{t}^{k} & x(M,2{)}_{t}^{k} & \cdots & x(M,N{)}_{t}^{k} \end{array}\right]$$

For the sake of reducing the impact of potential noise on the model prediction results, we utilized wavelet transform and a hard threshold function for denoising and smoothing each IMF. Finally, the values of the array were fed into the BiLSTM model for prediction.

#### 3.3.2. Bidirectional LSTM (BiLSTM Model)

LSTM has emerged as a dominant deep learning neural network and is being extensively employed in different domains including traffic flow classification, abnormal trajectory detection, and traffic prediction. LSTM [25] is a subtype of Recurrent Neural Networks (RNNs) that offers both long-term and short-term memory capabilities for making future predictions. One of the key features of LSTM is its ability to adapt and modify the assigned weights during the learning process. This adjustment is achieved using forget gates *f_t_*. Given an input sequence x = (x_1_, x_2_, …, x_T_) and LSTM memory *h_t_* at time *t*, the forget gate utilizes a *sigmoid* function σ to make a decision using *f_t_* at time *t*. The *sigmoid* function is used to calculate *f_t_* is shown below [25]:

*f_t_* = σ(*w_fh_*[*h_t_*_−1_], *w_fx_*[*x_t_*],*b_f_*) (8)

Furthermore, in addition to the forget gates, LSTM employs two additional gates known as the input and output gates. The input gate, denoted as *i_t_*, determines whether new information should be added to the memory cell. The input gate consists of two functional layers: a *sigmoid* function layer, which decides whether the cell weights should be updated, and a *tanh* function layer, which generates a vector of candidate values $\tilde{c_{t}}$ to be combined with the memory cell. The values of *i_t_* and $\tilde{c_{t}}$ are computed as follows:

*i_t_* = σ(*w_ih_*[*h_t_*_−1_], *w_ix_*[*x_t_*],*b_i_*) (9)

(10) $$\tilde{c_{t}}=tanh(w_{ch}[h_{t-1}],w_{cx}[x_{t}],b_{c})$$

The value in the memory cell can be updated using the vector $c_{t}$, which is calculated based on the following Equation [25]:

(11) $$c_{t}=f_{t}*c_{t-1}+i_{t}*\tilde{c_{t}}$$

where $c_{t-1}$ is the previous vector.

The output gate plays a crucial role in determining whether a value in the cell should be passed to the output. It makes this decision by utilizing a *sigmoid* layer denoted as *o_t_*, and is calculated according to Equation (12) [25].

(12) $$o_{t}=\sigma (w_{\mathrm{oh}}[h_{t-1}],w_{\mathrm{ox}}[x_{t}],b_{o})$$

Furthermore, the output gate maps the values within a range of [−1, 1] using the *tanh* function *ht*, which can be expressed using Formula (13):

(13) $$h_{t}=o_{t}\;*tanh(c_{t})$$

In all of the aforementioned equations, *b_f_*, *b_i_*, *b_c_*, and *b_o_* represent the biases, while *w_fh_*, *w_fx_*, *w_ih_*, *w_ix_*, *w_ch_*, *w_cx_*, *w_oh_*, and *w_ox_* are the weight matrices involved in the calculations.

In this research, we employed a bidirectional LSTM network as the foundation for our proposed model. BiLSTM [26] is an extension of LSTM that enables computation in the forward and backward directions.

**Figure 11:**
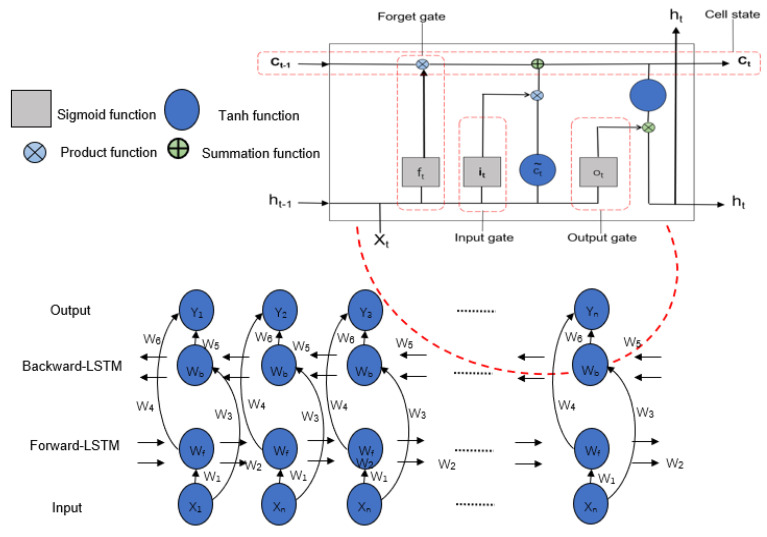
Architecture of bidirectional LSTM network.

In BiLSTM, LSTM neurons are divided into separate forward and backward states, which allows the network to capture information from both past and future instances, as the forward and backward hidden layers connect to the same output. Consequently, the utilization of BiLSTM enabled our model to achieve more accurate predictions by learning from both preceding and subsequent values and their corresponding weights. Figure 11 illustrates the key features of a BiLSTM network.

### 3.4. Model Implementation

**Figure 12:**
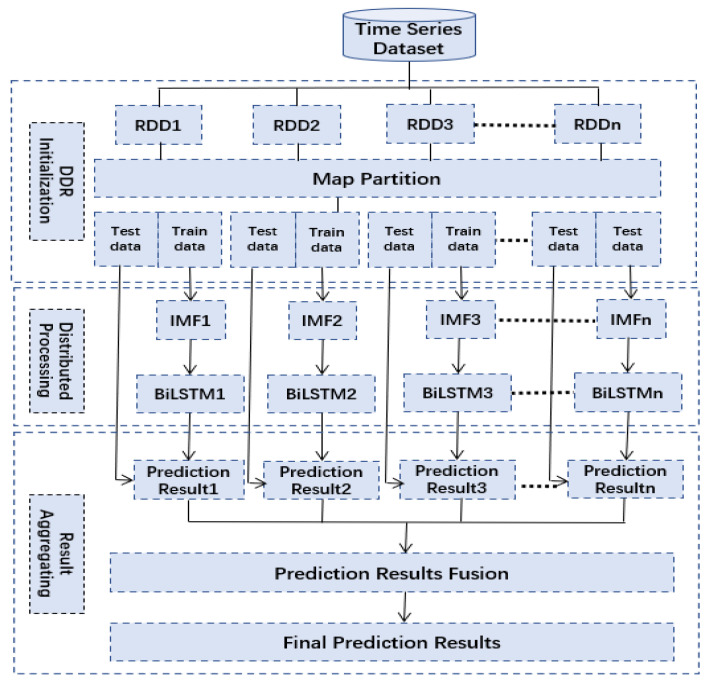
Distributed implementation of VMD-BiLSTM model on Spark.

To improve the scalability and the efficiency of taxi demand prediction, the proposed VMD-BiLSTM was applied on the Hadoop distributed data storing platform, utilizing the Spark distributed processing framework. The implementation of the distributed VMD-BiLSTM model consists of three main steps, namely Resilient Distributed Dataset (DDR) Initialization, Distributed Processing, and Results Aggregation. The distributed implementation of the VMD-BiLSTM model is illustrated in Figure 12.

This distributed approach allows for parallel processing and distributed storage of data, enabling the model to handle large-scale datasets and perform computations in a more efficient manner. By leveraging the capabilities of Hadoop and Spark, the VMD-BiLSTM model can effectively address the computational challenges associated with short-term taxi demand prediction.

#### 3.4.1. DDR Initialization

In this study, the taxi demand dataset was structured using time series data. For the sake of facilitating data processing and analysis, the SparkContext *partitionBy*() function was employed.

The *partitionBy*() function enables the time series data to be efficiently transferred and partitioned into multiple RDD partitions. Each RDD partition is then mapped with key–value pairs to facilitate subsequent operations. As part of the preprocessing stage, a map partition function is applied to each RDD partition, allowing for the partitioning of the data into training and testing subsets, which are transferred to be computed by the distributed VMD-BiLSTM model. Therefore, time series data can be effectively distributed and processed in parallel, optimizing the computational efficiency and scalability of the VMD-BiLSTM model for taxi demand prediction.

#### 3.4.2. Distributed Processing

The VMD-BiLSTM model was designed to perform short-term taxi demand prediction. To train the proposed VMD-BiLSTM model, the training data were processed independently within each RDD partition, utilizing Equations (2)–(13). Once the model was trained, it was validated using the testing data. During the validation process, the model generated local prediction results, which were then stored locally within the respective RDD partitions. This approach allowed for efficient and distributed prediction, as the computations and storage of prediction results were performed within the same partitions where the data resided. By leveraging the distributed computing capabilities of the RDD partitions, the VMD-BiLSTM algorithm can effectively generate accurate short-term taxi demand predictions.

#### 3.4.3. Results Aggregating

The local prediction results generated by the VMD-BiLSTM model were combined to produce the global prediction results. The aggregation process involved combining the predictions of each RDD partition to generate a final prediction for the entire dataset. This step was critical to ensure accurate and consistent results across the entire dataset. Finally, the forecasting results were outputted for use in real-world applications such as taxi dispatch and resource allocation. By utilizing the distributed computing capabilities of Hadoop and Spark, the VMD-BiLSTM algorithm can process large volumes of data efficiently and provide accurate predictions in real time.

## 4. Experimental Design

### 4.1. Data Preparation

The trajectory dataset of the target area of Wuhan City’s taxis was used in the experiments for this study. The dataset included key spatiotemporal attributes and characteristics such as Timestamp Longitude, Latitude, Speed, Direction, Taxi Status of Pickups, and Drop-offs. In addition, by analyzing the main features and by extracting spatiotemporal correlations, the taxi demand distribution of Wuhan city areas was obtained and more information was examined such as the peak travel period on days, weekdays, and weekends concerning taxi demand; crowded areas; crowded periods; etc. These obtained data were valuable in setting the prediction scenarios and forecasting results.

To investigate the performance of our prediction model on datasets of various sizes, the experimental data were divided into seven groups. These groups corresponded to different periods: 1 day, 1 week, 2 weeks, 1 month, 2 months, and the entire dataset spanning four months. This division allowed for a comprehensive evaluation of the models’ performance under varying dataset sizes.

In the extensive experiments, 70% of the dataset was used as the training dataset, while the remaining 30% served as the testing dataset. This split ensured that the models were trained on a significant portion of the data while still having sufficient data for evaluation. To analyze a wider range of high-resolution GPS locations and timestamps, taxi drop-off and pickup events were both considered taxi demands [22,27,28]. By considering both events, a more comprehensive understanding of taxi demand patterns could be achieved.

Following [27], the dataset of the study was divided into a training set and a test set. Detailed information on the dataset division is illustrated in Table 3.

### 4.2. Baselines

To compare the efficiency of our proposed model with state-of-the-art models, four forecasting models were used. These models included both non-distributed and distributed models. The non-distributed models used for comparison were GAN_CNN_LSTM [22] and MLRNN [16]. The distributed models chosen for comparison were grid-search-based SVM [17] and EMDN-GRU [18].

(1) GAN_CNN_LSTM [22] combines Generative Adversarial Network, Long Short-Term Memory, and Convolutional Neural Network models for performing taxi demand prediction. The model considers the benefits of using CNNs and LSTM to capture the main factors and then increase the accuracy of taxi demand prediction. The parameter settings are as follows: the generator uses four layers of LSTM; the neurons are 1024, 512, 256, and 128; 3 fully connected layers are implemented; and an ReLU is the activation function. The discriminator consists of four convolutional layers of 32, 64, 128, and 256 neurons. Moreover, a layer is integrated to produce a feature vector to be flattened, followed by four fully connected layers, and the output layer utilizes sigmoid as the activation function.
(2) The MLRNN [16], which stands for Multi-Layer RNN, utilized pairwise clustering and an RNN and considered the correlations among various areas for time series forecasting. In the MLRNN, the Adam optimizer is applied, and 0.001 is selected as the learning rate value. The input dimension of MLRNN is set to 8 × 2 × N, whereas the dimension of output is set to 2 × N, and the external factors dimension is set to 20 along with a fully connected layer with an ReLU as the activation function [16].
(3) Grid-SVM [17] utilizes a Support Vector Machine (SVM) algorithm with a grid search approach to forecasting passenger hotspots. A grid search-based SVM gridded the urban traffic road network on the Spark processing platform. For the parameter tuning in the grid search-based SVM, the selected parameter combination is C = 900 and γ = 0.001, as in [17], and the model is implemented on Spark with RDD.
(4) EMDN-GRU [18] stands for Empirical Mode Decomposition-based Network with Gated Recurrent Unit, which combines the EMD technique with a GRU neural network for prediction. EMDN-GRU utilizes the Spark platform to increase the speed of forecasting passenger waiting time on weekends and weekdays. For the parameter tuning in EMDN-GRU, the batch size and epoch are set to 4 and 180, respectively; the number of neural network layers and the number of neurons are set to 2 and 432, respectively. Our model was conducted on Hadoop with Spark processing framework [18].

As selecting the optimal hyperparameters for the proposed model can play a significant role in the performance of the proposed network, three parameters were considered, namely, the number of hidden units of the LSTM layers, the number of training epochs, and the dropout rate. Initially, we indicated a range of values for each parameter and then, during the training process, the model’s performance was explored and analyzed to specify the optimal parameters of the model. To be more specific, the number of hidden units of each LSTM layer was 8, 12, 16, 32, 64, and 128, whereas the dropout value ranged between 0.2 and 0.5, and the epochs ranged from 50 to 500.

Therefore, the optimal parameter settings of our model were one input layer with six dimensions followed by 2 LSTM hidden layers including 12 dimensions. Moreover, one dropout layer containing 12 dimensions was utilized where the value of dropout rate was 0.3, and finally, one dimension output layer was applied along 24 batch sizes, and the epochs were 400.

Additionally, the first two non-distributed models achieved significant results in taxi demand prediction but ignored the issue of dealing with large sample data, which can require more time consumption and memory cost and high I/O; these issues influence the stability and the efficiency of the prediction process. The last two predictive models provided significant prediction results, but more optimal methods for the learning process may need to be proposed for short-term taxi demand prediction.

By including these four models in the comparison, we can evaluate the performance of our model against similar model approaches, both in terms of non-distributed and distributed models.

### 4.3. Experimental Settings

In this study, the Wuhan City dataset was used for the experiments. The Wuhan trajectory dataset required data preprocessing from the beginning. This allowed us to collect larger datasets, which ultimately improved the performance of the prediction models.

The experiments were conducted using the Spark platform. The Spark platform cluster consisted of one master node accompanied by seven slave nodes. The master node was equipped with 8 gigabytes of RAM, 80 gigabytes of storage, and an eight-core processor. The slave nodes had 4 gigabytes of RAM, 40 gigabytes of storage, and 10-core processors. The experimental environment included Ubuntu 18.4, Hadoop 3.1, Spark 3.2, Python 3.7.0, and Pycharm 3.3 programs, as well as Anaconda 3.5, Pyspark 2.4.8, Vmdpy, SkLearn, and TensorFlow.

It should be noted that the experimental settings for the compared models were based on the information provided in the related articles or inferred from the available code details. However, the implementation of the models was challenging as many parameters and implementation details were not explicitly mentioned in the articles. Some of these details had to be guessed to reproduce the experimental results successfully.

### 4.4. Evaluation Metrics

For the aim of analyzing the performance of the distributed VMD-BiLSTM, we use three popular metrics: MAPE (mean absolute percent error), RMSE (root mean square error), and MAE (mean absolute value) as the measures of effectiveness (MOEs). These metrics are generally used to evaluate the accuracy and precision of forecasting models. MAPE is a percentage indicating the average deviation of the predicted values from the actual values, whereas RMSE is the square root of the mean of the square of all of the errors between actual values and predicted values. MAE is the difference between the predicted values and actual values [29].

These indices are commonly used in the field of data forecasting [30]. The values of MAE, MAPE, and RMSE can be obtained as follows:

(14) $$\mathrm{MAPE}=\sum_{t=1}^{n}\frac{\left|X_{t}-\hat{X_{t}}\right|}{X_{t}}\times 100\%$$

(15) $$\mathrm{RMSE}=\sqrt{\frac{1}{n}\sum_{t=1}^{n}{\left(X_{t}-\hat{X_{t}}\right)}^{2}}$$

(16) $$\mathrm{MAE}=\frac{1}{n}\sum_{t=1}^{n}\left|X_{t}-\hat{X_{t}}\right|$$

where $X_{t}$ is the actual value of taxi demands in a specific area at time t; $\hat{X_{t}}$ is the predicted value of taxi demands in the same area and time, and n represents the processed taxi demands in the given time interval.

To assess the efficiency and scalability of the proposed model, the running time and scalability are examined. This analysis helps determine how well the distributed VMD-BiLSTM model performs in terms of execution time and scaleup metrics.

In our study, another important metric that we examined was speedup. Speedup allowed us to evaluate the speed at which the distributed model performed compared to the corresponding sequential models. It provides insights into the efficiency gained through distributed processing. Speedup is defined as the ratio between the best sequential execution time and the parallel execution time. Speedup is a function of the parallelism degree of the parallel execution. The formula for calculating speedup is as follows:

(17) $$\mathrm{Speedup}=\frac{T_{seq}}{T_{par(n)}}$$

Here, *T*_seq_ represents the sequential execution time of the target model on a single node using the target dataset, whereas $T_{par(n)}$ is the distributed required time for tackling a similar prediction problem on a cluster consisting of n nodes [21]. By comparing these execution times, we can determine the speedup achieved by the distributed model. A higher speedup value indicates improved efficiency and faster execution of the model on the distributed platform.

In addition to the speedup of the calculations, we also considered the Scaleup metric to evaluate the performance of the parallel algorithm during handling larger datasets as the number of nodes increased.

Scaleup is the ratio between the parallel execution time with a parallelism degree equal to l and the parallel execution time with a parallelism degree equal to n. Scaleup measures the ability of the distributed model to effectively process larger volumes of data. The formula for calculating scaleup is as follows:

(18) $$\mathrm{Scaleup}=\frac{T_{par(1)}}{T_{par(n)}}$$

where $T_{par(1)}\;$ is the parallel execution time with parallelism degree equal to 1. By comparing these execution times, we can assess how well the parallel algorithm scales with an increasing number of nodes.

A higher scaleup value indicates that the distributed model can efficiently handle larger datasets and effectively leverage the resources provided by the cluster. This metric is essential for evaluating the scalability and performance of the distributed model.

## 5. Results and Analysis

The specifications of the proposed model and the other models were applied as given in Section 4.2. Then, the models’ results and performance were examined using the metrics defined in Section 4.4. In this section, the prediction results are explored and analyzed by considering the advantages of applying the methods in the proposed model. Additionally, the experimental results are compared across the predictive models listed in Section 4.2.

### 5.1. Taxi Demand Prediction

**Figure 13:**
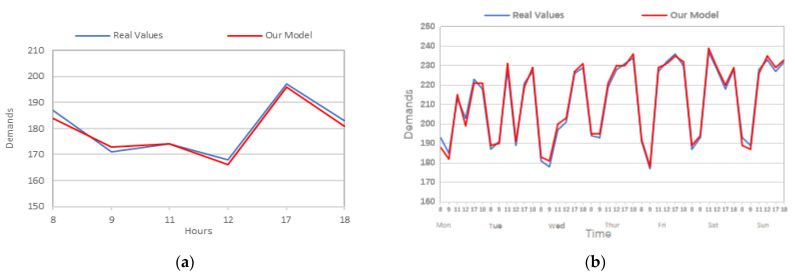

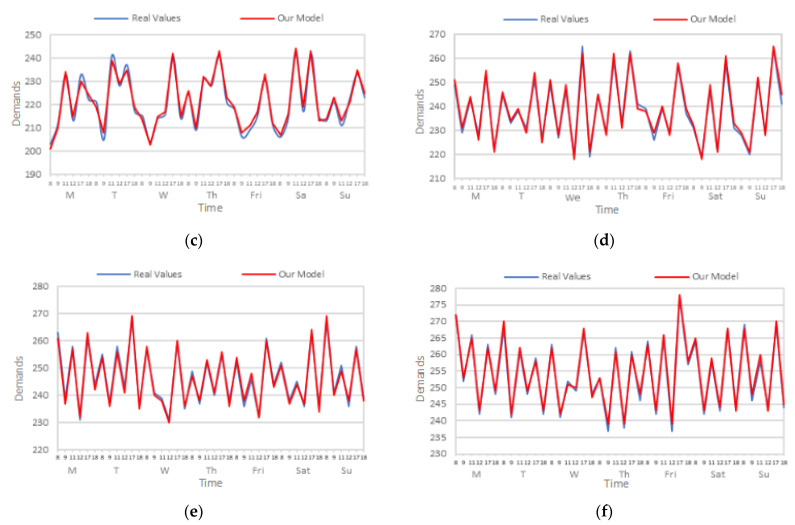
Results of our prediction model on Wuhan’s dataset. (**a**) 1 day; (**b**) 1 week; (**c**) 2 weeks; (**d**) 1 month; (**e**) 2 months; and (**f**) whole dataset.

For the sake of comparing the actual taxi demand values with the predicted values obtained by our model, Figure 13 visualizes the main features of the results of the prediction model on the Wuhan dataset. Due to this, the prediction values are massive and it becomes hard to apply a comparison of the actual values with the predicted values for the whole set. Therefore, the average numbers for the periods of 8:00, 9:00, 11:00, 12:00, 17:00, and 18:00 for both actual and predicted values are considered and visualized in the following subfigures.

Figure 13 presents a comparison of the prediction values vs. the real values by only considering the peak hours (8:00, 9:00,11:00, 12:00, 17:00, and 18:00) among different data sizes (1 day, 1 week, 2 weeks, 1 month, 2 months, and whole dataset). We can see that the predicted values are almost the same as the actual values and both are special on big-size datasets. The average values gradually increase with the rising of the volume of the datasets; however, the two values are close and similar sometimes, especially in Figure 13d–f.

### 5.2. Ablation Study

To validate the efficiency of the integrated methods (VMD and BiLSTM) utilized in our model, we removed the VMD method from the model, replacing VMD with EMD and replacing BiLSTM with LSTM and GRU, respectively, and conducting the experiments by considering the same dataset and keeping the other hyperparameters unchanged and then we examined the performance of the models. Additionally, for the sake of verifying the advantages of the distributed models, we compare the performance of the non-distributed VMD BiLSTM and distributed VMD BiLSTM models proposed in this study.

Table 4 shows the results of prediction performance (average of accuracy metrics for the predictions of at the peak hour of 8 am) of the four models: our model without VMD, EMD_BiLSTM, VMD_LSTM, VMD-GRU, and our model using the Wuhan dataset.

The experimental results indicate that our model (integrating VMD and BiLSTM methods) outperforms the other three degenerate models. In other words, adding the VMD and BiLSTM methods remarkably influenced the prediction performance of the forecasting model. Furthermore, the non-distributed VMD-LSTM model performs worse than the distributed VMD-LSTM model.

The models without VMD performed the worst in all predictions and the EMD_BiLSTM model ranked the second worst among the models, which indicates that the VMD method has the greatest impact on prediction accuracy. The reason behind this may be that since the essence of taxi demand data are time series data, the features of the sequence itself are the most important and VMD can effectively extract the features of the time series dataset, which in turn enhances the forecasting performance.

From Table 4, comparing with the results of our model, we find that the models without the BiLSTM method present slightly decreasing accuracy, highlighting that adopting BiLSTM assists in effectively learning the main features in the dataset and reducing the loss accuracy, as BiLSTM can learn new knowledge from the features by conducting learning process in both the forward and backward directions.

### 5.3. VMD Results

**Figure 14:**
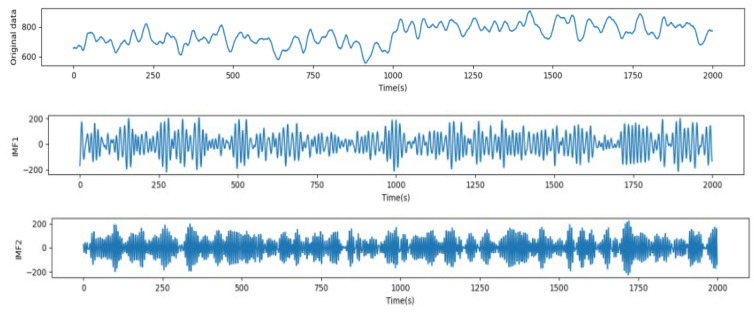

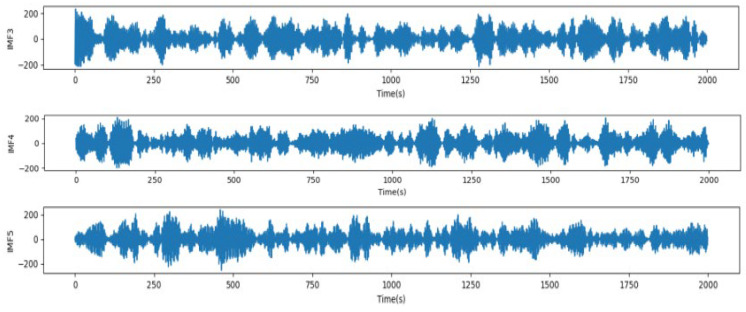
VMD renderings.

Before performing VMD on the original taxi demand sequence, the optimal values of the decomposition mode K, the quadratic penalty factor α, and the convergence tolerance τ need to be determined. By applying the grid search method and exploring the MSE results during different combinations of these parameters within specified ranges, the experimental tests show that when K > 3, the center frequencies of the subsequences starting from the third layer are very similar, and it is determined that over-decomposition occurs; when K = 3, α = 1500, and τ = 1 × 10^−4^, we can obtain the best results of VMD. The original load sequence and its subsequence effect obtained by VMD are shown in Figure 14.

For the sake of experimentally demonstrating the correction of selecting the number of the subsequent subsequences produced by VMD, we experiment with the proposed model for exploring the prediction results of the center frequencies of the subsequent subsequences, and the results are shown in Table 5. The experiment was performed on the whole dataset and the prediction horizon period was 5 min.

The data listed in Table 5 demonstrate that the VMD-BiLSTM model attains good results for the forecasting for each VMD component. Generally, the forecasting results of low- and medium-frequency components (IMF1 and IMF2) are more efficient; their R2 values are 0.995 and 0.993. The IMF3 component also generated good predictions; its R2 value is 0.991. On the other hand, with the high-frequency components IMF4 and IMF5, the prediction results decrease and become worse, with an R2 value of 0.983 and 0.975 for IMF4 and IMF5, respectively.

**Figure 15:**
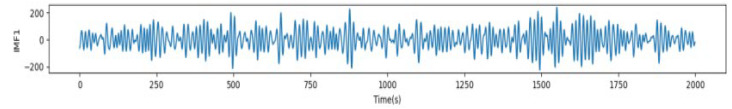

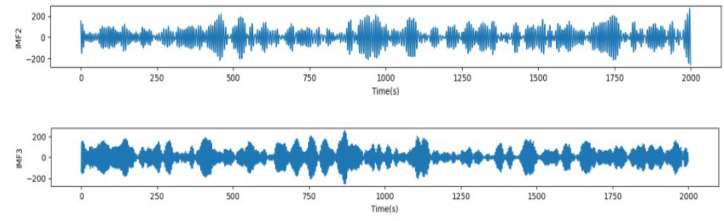
Wavelet threshold denoising method for VMD renderings (IMF1, IMF2, and IMF3).

By exploring the findings in Figure 12 and Table 4, we can conclude that it can be shown experimentally that the center frequencies after the third layer do not improve the accuracy of the results; rather, the accuracy values slightly fall. These findings support the decision we made to select K = 3 as the optimal selection for the number of the center frequencies of the subsequent subsequences. Additionally, for the sake of further reducing the impact of the noise on the prediction results, the wavelet transform was applied to denoise each IMF, and the denoising results are illustrated in Figure 15.

It is obvious that the data curve after applying the wavelet denoising becomes smoother and the data features are more distinguishable.

### 5.4. Temporal Prediction Horizon

To verify the impact of the temporal attributes on the predictions of the model, our study further divided the intervals of time into several smaller periods. We set the time intervals to 5, 10, 15, and 20 min. Table 6 depicts a comparison of the prediction results of several models for varied prediction horizons. The values of MAE, MAPE, and RMSE demonstrate that our model achieved superior performance compared to the other models in the 5 min period.

The results presented in Table 6 show that our model generated the smallest error among all predictions, as the VMD and LSTM methods have a better ability to map taxi demand data from the target study area and benefit the most from the spatial and temporal features for demand prediction, thereby resulting in perfect prediction performance. In addition, when we consider the 5 min time interval as the essential period, the MAE, MAPE, and RMSE had the smallest values by remarkable percentages as well. Therefore, we utilized a 5 min interval as the main reference for the temporal prediction horizon for this study.

### 5.5. Distributed VMD-BiLSTM Results

In this study, we examined the taxi demands (pickups and drop-offs) in the selected areas during different periods and performed time series prediction, and then we explored the accuracy of prediction, loss function, running time, and scalability. The dataset was divided into different period-based sizes, including 1 week, 2 weeks, 1 month, 2 months, and the whole dataset (4 months), and the predictive models were applied to forecast the taxi demands for the coming periods and then we compared the results with the real taxi demands.

#### 5.5.1. Effectiveness Comparison

(1) Accuracy Comparison

For the sake of evaluating the accuracy of the proposed model, we conducted experiments using different subsets of the dataset. The dataset was divided into various time intervals, including 1 day, 1 week, 2 weeks, 1 month, 2 months, and the entire dataset. For each interval, 70% of the data were used for training the models, while the remaining 30% were used for testing the models.

**Figure 16:**
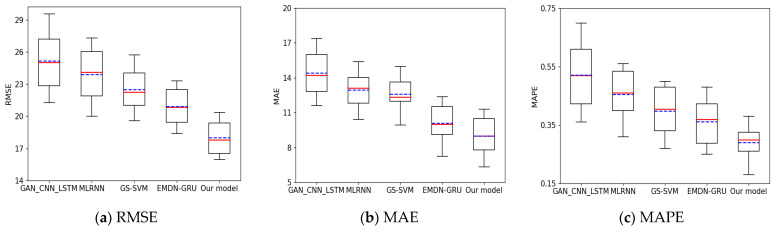
Box-plot of MOEs for Wuhan dataset.

**Figure 17:**
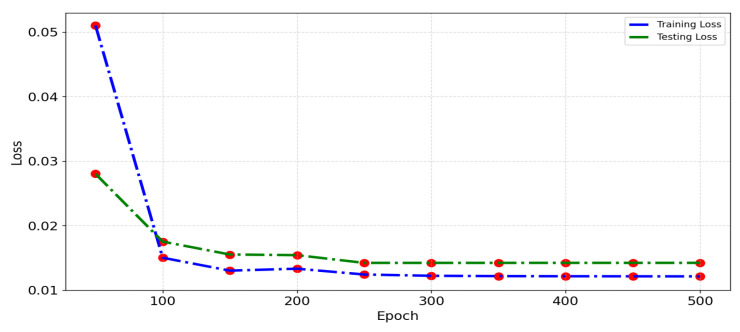
Comparison of loss function of distributed VMD-BiLSM.

**Figure 18:**
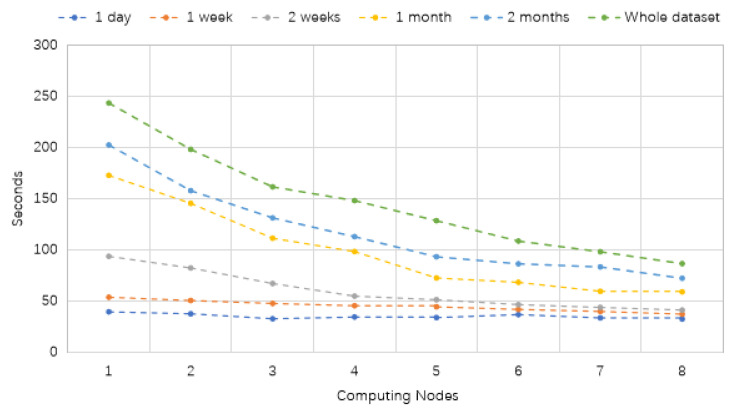
Running time (seconds) of VMD-BiLSTM based on Spark platform.

**Figure 19:**
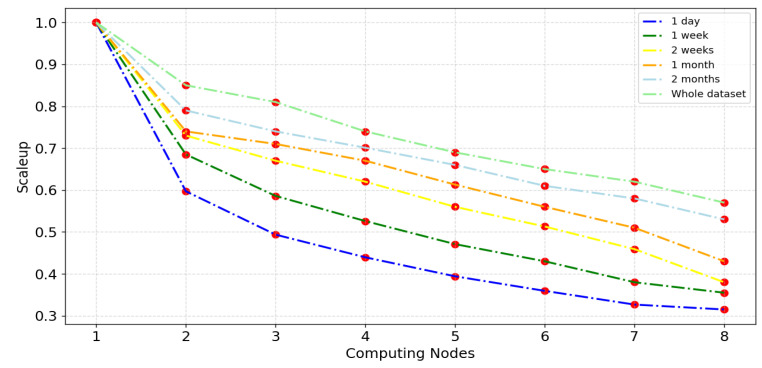
Scaleup comparative analysis of distributed VMD-BiLSTM for different computing nodes.

To evaluate the performance of the proposed model, we compare it against existing state-of-the-art models using the GPS dataset of Wuhan City, China. The comparison results are presented in Table 7. Additionally, we provide the experimental results obtained with different datasets, which are depicted in Figure 16, Figure 17, Figure 18 and Figure 19.

These comparisons and experimental results allow us to assess the average accuracy and the effectiveness of the distributed VMD-BiLSTM for forecasting taxi demands. The model’s performance can be analyzed across different time intervals, providing insights into its suitability for various prediction scenarios.

Applying Wuhan City’s GPS dataset, considering Table 7 and Figure 16, the MAPE values of the distributed VMD-BiLSTM model are significantly lower than those in GAN_CNN_LSTM [22] and MLRNN [16], GS-SVM [17], and EMDN-GRU [18]. The finding results for 1 day, 1 week, 2 weeks, 1 month, 2 months, and the whole dataset are illustrated in Table 7 and visualized by a box-plot chart, as shown in Figure 16.

When analyzing the Wuhan dataset, it is evident from Table 7 and Figure 14 that the distributed VMD-BiLSTM model outperforms the GAN_CNN_LSTM, MLRNN, GS-SVM, and EMDN-GRU models in terms of MAPE values. The MAPE values achieved by the distributed VMD-BiLSTM are considerably lower than those obtained by the other methods.

To further illustrate the experimental results with the Wuhan dataset, Table 7 provides a comprehensive overview of the model performance across different time intervals, including one day, one week, two weeks, one month, two months, and the entire dataset. The box-plots offer a concise summary of the model’s performance, showcasing the variability and distribution of the MAPE values for each time interval. When considering the entire dataset, our proposed model demonstrates significant improvements over other models in the obtained results for the MAPE, RMSE, and MAE. Specifically, our model achieves a MAPE value that is 52%, 43.2%, 44.95%, and 48.01% lower than that of GAN_CNN_LSTM, MLRNN, GS-SVM, and EMDN-GRU, respectively. This indicates a substantial reduction in the prediction error compared to the alternative models.

Furthermore, the RMSE values obtained by our proposed model exhibit notable reductions across all six groups of datasets, with improvements of 42.22%, 41.82%, 4.1%, and 39.93% compared to GAN_CNN_LSTM, MLRNN, GS-SVM, and EMDN-GRU, respectively. The results demonstrate the enhanced accuracy of our model in capturing the actual taxi demands. Analyzing the MAE values further reinforces the superiority of our proposed model. It achieves MAE values that are 62.3%, 31.12%, 24.87%, and 57.40% lower than GAN_CNN_LSTM, MLRNN, GS-SVM, and EMDN-GRU, respectively. This significant reduction in prediction errors demonstrates the effectiveness of our model in accurately estimating taxi demands.

Overall, the comparison of MAPE, RMSE, and MAE values clearly highlights the remarkable performance of VMD-BiLSTM across the entire dataset. It consistently outperforms other models, showcasing its effectiveness and accuracy in predicting taxi demands.

(2) Computation Speed Comparison

The computation speed was calculated and compared for VMD-BiLSTM and other prediction models on the dataset of Wuhan City. The process considers a five-minute prediction along with two perspectives: training and inference time. Our proposed model and the other prediction models are tested with similar conditions to obtain a fair comparison. The comparison results are illustrated in Table 8.

From the findings listed in Table 8, it can be noticed that:

- GAN_CNN_LSTM is associated with the minimum training time and the minimum inference time, but it obtained the lowest forecasting performance among all compared models (by MAE, MAPE and RMSE results in Table 4).
- MLRNN and GS-SVM have a slightly shorter training and inference time. It is relatively longer than GS-SVM. However, GS-SVM shows the second worst results among neural-aware models in Table 8.
- Both EMDN-GRU and our model adopted distributed computing prediction and applied decomposing algorithms (EMD and VMD), but our model is superior as it was around a four-times faster than EMDN-GRU when it came to training and inference.

#### 5.5.2. Loss Function Analysis

Figure 17 depicts the loss function for the distributed VMD-BiLSTM model when trained and tested on the whole Wuhan dataset within a five-minute period. The graph covers different epochs ranging from 50 to 500. The training and testing processes of the proposed model demonstrated noteworthy consistency between the training loss and the testing loss.

These results mean that the proposed model achieved a convergence without exhibiting significant overfitting.

By analyzing Figure 17, we find that training loss and testing loss are consistent, and the model converged without overfitting. The taxi demand prediction performance of the proposed model consistently outperforms other models, showcasing its effectiveness and accuracy in predicting taxi demands.

#### 5.5.3. Distributed Running Time Analysis

The running time of the proposed model has been evaluated across different numbers of computing nodes and various dataset sizes. The results are presented in Table 9 and Figure 18. Interestingly, it is observed that the running time of the distributed VMD-BiLSTM model decreases as the number of computing cluster nodes increases, both on single-node and multiple-node Spark clusters.

From Table 9 and Figure 18, we notice that especially for the small files (one day and one week), the total execution time is almost the same. Additionally, regarding massive files, although the running time for conducting traffic predictions begins with high costs (above 150 s), the required time consumption is reduced sharply with the rise in the number of computing resources from three to eight.

In other words, the distributed models using distributed processing platforms are associated with a reduced execution time for taxi demand prediction.

#### 5.5.4. Scalability Analysis

The scaleup metric serves to assess the performance of a target model when handling larger datasets in the presence of additional computing nodes (computing cluster sizes). To analyze the scalability metric of VMD-BiLSTM, experiments were conducted by gradually increasing the number of nodes, from one to eight active Spark slaves, as a direct proportion of the dataset sizes. The datasets used for this evaluation varied in size, ranging from smaller datasets (1 day and 1 week) to larger ones (2 months and the entire dataset). Each experiment was repeated three times to guarantee the accuracy of the findings. The results of these experiments are plotted in Figure 19 for the Wuhan dataset. The figure provides visual representations of the scaleup performance of the VMD-BiLSTM model under different dataset sizes and computing node configurations.

Figure 19 shows that as the number of nodes increases, scaleup values sharply decrease in the range [0.85–0.6] at the second slave node because, in the proposed model building process, the training process in the first node is conducted serially. Moreover, the scaleup ratio gradually decreases from the second slave node to the eighth node where the value ranges are 0.31–0.58. It is evident that all the scaleup values of the VMD-BiLSTM model exceed 0.31, showcasing a proportional increase in the size of datasets and the number of computing nodes. This indicates that our model can properly utilize the additional processing power and distribute the workload across the processing nodes, which enhances the performance of the forecasting results. This finding suggests that the VMD-BiLSTM model exhibits excellent scalability on the Spark cluster and possesses the ability to handle large datasets effectively.

**Figure 20:**
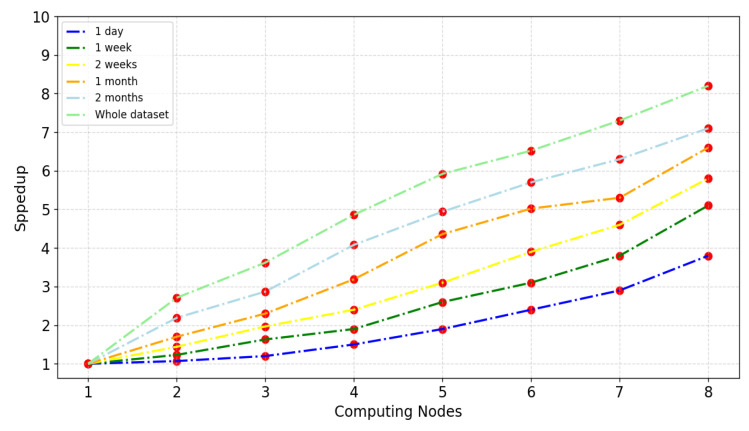
Speedup comparative analysis of the proposed model for different computing nodes.

To analyze the speedup performance of the VMD-BiLSTM model, we conducted experiments using a cluster with a varying number of nodes, ranging from one to eight (equivalent to 4 to 32 cores), while considering six different data scales: 1 day, 1 week, 2 weeks, 1 month, 2 months, and the entire dataset. The experimental results are shown in Figure 20, which provides insights into the speedup achieved by the VMD-BiLSTM model under different node configurations and data scales.

From Figure 20, it is evident that the speedup of the VMD-BiLSTM model exhibits a relatively linear increase as the number of computing nodes grows. Notably, larger datasets tend to achieve a better speedup. In particular, the speedup value with the largest amount of data reaches 8.123, which is close to the ideal speedup, when utilizing eight nodes. Achieving linear speedup is challenging due to factors such as communication costs and imbalances among the slave nodes [31]. Furthermore, the results highlight that as once data size gradually increases, the computation time becomes remarkably dominant.

## 6. Discussion

In this study, our results reveal that the use of a VMD method associated with bidirectional LSTM enhances the effectiveness and the scalability of forecasting taxi demands. Once the time series data are decomposed into Intrinsic Mode Functions, the main features are properly extracted and the non-linearity issue is solved, which reduces the huge time consumption required for processing short-term predictions. Additionally, the findings indicate that the BiLSTM model plays a vital role in the forecasting process due to its incredible power in capturing both backward and forward features.

The present findings align with those of Xia et al. [14,18], who observed the significance of using the EMD method in traffic prediction. However, VMD, which is used in our study, is a more efficient method than the EMD algorithm, since it is capable of decomposing a given signal into lesser modes with non-recursive iteration. 

Our study runs contrary to the studies [9,18], which address the issue of taxi demands by applying deep learning-based methods such as traditional LSTM and GRU. However, our study introduces BiLSTM as it is able to process the input in both directions and capable of utilizing information from both sides, which in turn remarkably increases accuracy.

The experiment provides new insight into applying big data techniques and disturbed platforms to reduce the computation time during the forecasting process. While previous research [18,21] has focused on utilizing standalone or MapReduce, the results illustrated by Table 9 and Figure 18, Figure 19 and Figure 20 demonstrate the results of adopting Spark instead. This can be interrupted as Spark uses RAM (random access memory) to process data and stores intermediate data in memory, which reduces the amount of read and write cycles on the disk. This makes it faster than MapReduce. Moreover, the various parameters such as the number of cluster nodes and the data size can directly influence the stability and scalability of the traffic forecasting results.

While this study highlights valuable insights regarding enhancing taxi demand prediction, it is essential to acknowledge some limitations. Firstly, there is a need to investigate the effectiveness of adding other variables, such as weather information, to the taxi demand prediction model. Then, prediction results can be enhanced and lead to obtaining a more comprehensive understanding of taxi demands. Another limitation is that using a real-time dataset can improve traffic predictions because some areas are associated with large demands and taxi drivers can compete to find passengers in the other areas. Such issues need to be addressed by providing a more comprehensive understanding of taxi flow and demands. The model can be applied to real taxi demands by considering and adding real-time processing techniques—specifically, multi-source data collection, and real-time processing. Regarding data collection, there is a need to integrate Apache Kafka to collect and store streaming data that can easily fit within distributed applications, and can deal with billions of streamed data per minute. Additionally, for the sake of conducting real-time processing with deep learning models for taxi demand prediction, we can incorporate higher performance infrastructure such as GPUs and platforms for processing and analyzing data in real-time, such as Apache Flink.

In summary, as confirmed by the previously described findings, we are able to deduce that the distributed VMD-BiLSTM model achieves more accurate results than the comparable approaches for traffic prediction in the short term, providing valuable insights into the significance of adopting models that integrate decomposing algorithms and deep learning models along with distributed computing platforms such as Spark.

## 7. Conclusions

As traffic demand prediction becomes increasingly challenging for taxi drivers’ everyday lives, it is important to address growing non-stationary issues, high time consumption, and prediction accuracy when dealing with big traffic flow data. In this article, a novel distributed VMD-BiLSTM model was proposed to perform taxi demand prediction using large datasets. Then, taxi demand data were decomposed using the VMD method to generate Intrinsic Modal Functions (IMFs), thereby reducing the non-stationarity of the taxi demand data. Bidirectional LSTM (BiLSTM) was utilized to perform the forecasting process as its ability to capture both backward and forward features resulted in a remarkable increase in the overall model accuracy. The proposed model was deployed on Apache Spark to conduct time series predictions of big industry data utilizing parallelization technology. In the Section 4, we used a real taxi dataset to test the performance of the proposed model. The final results show that our proposed model outperforms cutting-edge prediction models in terms of accuracy metrics, running time, and scalability with remarkable improvement in computational efficiency.

In future work, we aim to utilize new deep learning architectures such as Mamba to better extract the main features of traffic demands and to achieve higher prediction accuracy. Additionally, incorporating visual features obtained from road cameras and other data resources into the dataset can enhance the prediction model as well.

## Figures and Tables

**Table 1:** Sorting records of taxi GPS sensor data in Spark.

(a) Timestamp-aware Sorting						
**TaxiID**	**Timestamp**	**Latitude**	**Longitude**	**Speed**	**Direction**	**Taxi Status**
2618113	201309010000100	30.621755	114.300586	35.335000	215.19	1
3175820545	201309010000100	30.552416	114.282483	88.985001	302.8	1
3175942145	201309010000100	30.607883	114.281766	60.495001	289.64	3
3175737345	201309010000100	30.543016	114.30915	58.830001	267.38	1
3176131841	201309010000100	30.547966	114.299416	72.519998	277.46	3
3175694081	201309010000100	30.577666	114.188066	0	123.97	1
3175821825	201309010000100	30.527366	114.354716	77.884999	289.75	1
3175907073	201309010000100	30.4715	114.125083	0	85.98	1
(b) Taxi ID-aware Sorting						
**TaxiID**	**Timestamp**	**Latitude**	**Longitude**	**Speed**	**Direction**	**Taxi Status**
2618113	20130901000100	30.621755	114.300586	35.335000	215.19	1
2618113	20130901000140	30.619966	114.299045	39.275503	211.79	1
2618113	20130901000219	30.619936	114.29576	57.1095	301.92	1
2618113	20130901000300	30.62314	114.28943	61.050002	301.82	1
2618113	20130901000340	30.62719	114.283288	52.558502	323.31	1
2618113	20130901000421	30.626928	114.282025	56.869002	245.72	1
2618113	20130901000540	30.624216	114.271246	23.402499	252.68	1
2618113	20130901000612	30.622628	114.270796	0	0	1

**Table 2:** Specifications of a trip.

Specifications	Explanation
Trip ID	Trip Identifier including Taxi ID
Trip Beginning Time	Pickup Timestamp
Trip Beginning Longitude	Pickup Longitude for a cruising trip
Trip Beginning Latitude	Pickup Latitude for a cruising trip
Trip Terminating Time	Drop-off Timestamp
Trip Terminating Longitude	Drop-off Longitude
Trip Terminating Latitude	Drop-off Latitude
Trip Distance	Total cruising distance from Pickup to Drop-off
Average Speed	Average Speed of the cruising trip
Cruising Time	Difference between Trip Terminating Time and Beginning Time

**Table 3:** The training and test distribution of the experimental dataset.

Area	Collection Period	Trip Whole Dataset		Training Set		Test Set	
Wuchang District	1 September 2013–31 December 2013	Size	Records	Size	Records	Size	Records
		200 GB	1,421,935	141 GB	995,355	59 GB	426,580

**Table 4:** Results of ablation experiments.

Dataset	Metric	Models					
		Our Model (without VMD)	EMD_BiLSTM	VMD_LSTM	VMD-GRU	Our Model (Non-Distributed)	Our Model (Distributed)
Whole dataset	RMSE	23.01	21.63	20.32	18.39	17.9	**15.82**
	MAE	13.93	11.74	10.19	8.32	9.49	**6.68**
	MAPE	0.46	0.41	0.31	0.21	0.23	**0.17**

The best results are marked in bold.

**Table 5:** Prediction results of VMD components.

Model	VMD Subsequent	RMSE	MAE	MAPE	R^2^
VMD-BiLSTM	IMF1	7.19	10.26	0.51	0.995
	IMF2	8.21	12.11	0.57	0.993
	IMF3	8.82	13.92	0.61	0.991
	IMF4	9.52	15.47	0.43	0.983
	IMF5	11.31	13.92	0.47	0.975

**Table 6:** Comparison of model prediction results for different prediction horizons.

	5 min			10 min			15 min			20 min		
	RMSE	MAE	MAPE	RMSE	MAE	MAPE	RMSE	MAE	MAPE	RMSE	MAE	MAPE
GAN_CNN_LSTM [22]	15.32	17.49	0.89	19.32	18.37	0.93	20.31	21.35	0.95	23.57	24.46	0.96
MLRNN [16]	13.29	16.36	0.77	18.71	17.51	0.87	19.47	18.47	0.91	22.67	20.39	0.94
GS-SVM [17]	9.83	13.67	0.61	10.26	14.82	0.72	17.35	16.42	0.83	19.28	18.93	0.89
EMDN-GRU [18]	8.62	12.12	0.53	9.33	12.38	0.63	13.86	14.36	0.78	17.39	17.66	0.82
Our Model	7.19	10.26	0.51	8.83	11.34	0.59	12.65	13.74	0.69	14.25	16.58	0.76

**Table 7:** Average MOEs of GAN_CNN_LSTM, MLRNN, GS-SVM, EMDN-GRU, and proposed model.

Dataset	Metrics	Models				
		GAN_CNN_LSTM [22]	MLRNN [16]	GS-SVM [17]	EMDN-GRU [18]	Our Model
1 day	RMSE	29.54	27.31	25.71	23.31	20.35
	MAE	17.38	15.39	14.97	12.38	11.30
	MAPE	0.70	0.56	0.50	0.48	0.38
1 week	RMSE	27.78	26.53	24.34	22.90	19.74
	MAE	16.39	14.24	13.98	11.95	10.93
	MAPE	0.63	0.55	0.49	0.43	0.33
2 weeks	RMSE	25.46	24.59	23.09	21.33	18.31
	MAE	14.90	13.41	12.63	10.31	9.10
	MAPE	0.55	0.49	0.45	0.40	0.31
1 month	RMSE	24.61	23.61	21.44	20.37	17.25
	MAE	13.57	12.80	12.03	9.69	8.89
	MAPE	0.49	0.43	0.36	0.34	0.29
2 months	RMSE	22.25	21.34	20.86	19.11	16.31
	MAE	12.56	11.48	11.98	8.92	7.41
	MAPE	0.40	0.39	0.32	0.27	0.25
Whole dataset	RMSE	21.26	20.01	19.57	18.39	15.97
	MAE	11.61	10.41	9.93	7.25	6.34
	MAPE	0.36	0.31	0.27	0.25	0.18

**Table 8:** Results of computation efficiency.

Dataset	Metric	Models				
		GAN_CNN_LSTM [22]	MLRNN [16]	GS-SVM [17]	EMDN-GRU [18]	Our Model
1 day	Training Time (s/epoch)	9.54	10.13	12.54	13.31	11.35
	Inference (s)	9.12	10.81	11.72	12.08	11.21
1 week	Training Time (s/epoch)	12.92	13.05	12.04	14.84	11.35
	Inference (s)	10.82	11.31	13.30	13.70	12.46
2 weeks	Training Time (s/epoch)	15.31	16.32	14.39	15.63	16.46
	Inference (s)	11.81	13.72	12.38	14.78	15.31
1 month	Training Time (s/epoch)	17.83	18.26	19.34	20.21	21.81
	Inference (s)	12.57	15.63	14.43	17.24	18.72
2 months	Training Time (s/epoch)	20.28	21.22	22.25	29.81	25.18
	Inference (s)	13.22	18.44	17.20	28.07	20.89
Whole dataset	Training Time (s/epoch)	34.89	35.98	39.48	44.72	40.31
	Inference (s)	18.28	27.41	26.93	37.25	28.92

**Table 9:** Running time (seconds) of VMD-BiLSTM based on Spark platform.

	Computing Nodes							
Dataset	1	2	3	4	5	6	7	8
1 day	39.39	37.52	32.65	34.34	33.78	36.72	33.49	32.39
1 week	53.68	50.47	47.59	45.38	44.31	41.78	39.82	37.18
2 weeks	93.63	82.29	67.1	54.77	51.27	46.48	43.72	41.16
1 month	172.77	145.44	111.38	98.31	72.51	68.25	59.47	58.92
2 months	202.45	157.82	131.21	112.88	93.19	86.35	83.26	72.22
Whole dataset	243.51	198.22	161.53	148.12	128.45	108.56	98.12	86.54

## Data Availability

The data presented in this study are available on request from the corresponding author.
